# Implementation and Evaluation of Peer Ambassador Support to Patients Newly Diagnosed With Hematological Cancer and Their Caregivers

**DOI:** 10.1155/nrp/4528051

**Published:** 2025-04-28

**Authors:** Kristina Holmegaard Noerskov, Iben Husted Nielsen, Emma Skov Rahbæk, Sarah Halbro, Astrid Østergaard Mortensen, Ulrik Malthe Overgaard, Karin Piil, Mary Jarden

**Affiliations:** ^1^Department of Haematology, Copenhagen University Hospital, Rigshospitalet, Copenhagen, Denmark; ^2^Department of Haematology, Zealand University Hospital, Roskilde, Denmark; ^3^Department of Oncology, Centre for Cancer and Organ Diseases, Copenhagen University Hospital, Rigshospitalet, Copenhagen, Denmark; ^4^Department of People and Technology, Roskilde University, Roskilde, Denmark; ^5^Department of Clinical Medicine, University of Copenhagen, Copenhagen, Denmark

**Keywords:** evidence-based nursing, experienced-based psychosocial, hematology, implementation science, peer support, psychosocial intervention, social support

## Abstract

**Aims and Objectives:** To provide a comprehensive report on the development and implementation of peer ambassador support to patients newly diagnosed with hematological malignant disease and their caregivers within a clinical setting.

**Methods:** The continued development and implementation of the peer ambassador support program took place between October 2022 and January 2024 at the Department of Hematology, Copenhagen University Hospital, Rigshospitalet. Grol and Wensing's 5-step model of implementation guided the process.

**Results:** This paper demonstrates the successful adaptation and implementation of the peer ambassador program and provides valuable insights into the enabling factors and obstacles encountered. In total, 48 peer ambassadors were recruited, and 27 patients and seven caregivers received support. Over 90% of patients and caregivers reported high satisfaction with the support, and the majority (70%) noted that the support improved their understanding and provided new insights into their disease and treatment journey.

**Conclusion:** The development and implementation of the intervention are complex and demanding long-term processes that require applying theoretical knowledge, understanding patient and caregiver's experiences, and collaboration with the interdisciplinary team in clinical practice.

**Implications:** This paper provides knowledge for implementing future peer ambassador support initiatives. It has potential transferability and value in a broader context for patients with life-threatening illnesses and their caregivers.

**Impact:** It contributes to research and transparency in the development and implementation of evidence-based nursing interventions. It confirms that implementation of a peer support intervention is complex and requires theoretical knowledge, application of that knowledge, and interdisciplinary collaboration in clinical practice. Finally, it provides evidence-based knowledge applicable to the implementation of future peer support initiatives.

**Patient Contribution:** Patients and their caregivers have been actively involved throughout the development and implementation of the peer ambassador support program.

**Trial Registration:** ClinicalTrials.gov identifier: NCT03493906 and NCT04039100.

## 1. Introduction

Hematologic malignant diseases are associated with high mortality and morbidity, and the disease trajectory is often characterized by an unpredictable, long-term clinical course [1]. Treatment is increasingly administered in the outpatient setting, involving prolonged high-dose combination chemotherapy and autologous or allogeneic hematopoetic stem cell transplantation (HSCT) with a high risk of complications, severe side effects, and a significant symptom burden [2, 3]. Receiving a diagnosis of an acute onset life-threatening hematological malignant disease can be emotionally challenging for both patients and their family caregivers [4]. Many patients, along with their family caregivers, often report elevated levels of psychological distress that adversely impact their quality of life (QOL) [3, 5–7]. Previous research has shown that patients undergoing treatment and their family caregivers have multiple information and support needs, including the need for experience-based psychosocial support [5, 8]. While each person experiences their cancer journey uniquely, many prefer to gain this additional support from peers with similar lived experiences [8–10].

Psychosocial support, such as peer support, presents a unique opportunity to offer support based on personal experiences, providing informational, emotional, and practical assistance beyond what health professionals and one's social network can provide [11]. The advantages of peer support in patients with cancer and family caregivers include improved psychological well-being, enhanced coping, increased self-efficacy, better QOL, hope, and improved adjustment, and it is considered a relevant approach in the field of patient education [12, 13]. In their role as peer support providers, peer ambassadors may also experience personal benefits, as helping others contributes to self-development and a sense of meaning, fostering a feeling of social usefulness [10, 14].

Previously, we conducted two studies investigating the feasibility of peer support interventions in newly diagnosed patients with acute leukemia (AL) [15] and family caregivers of newly diagnosed patients with hematological malignancies [16]. Both studies established the feasibility and safety of one-on-one peer support, providing evidence of the effectiveness of these interventions in supporting patients with hematologic malignancies and their caregivers.

Successful implementation of new knowledge in clinical practice is essential to ensure that patient care remains consistently updated, delivering high-quality healthcare. Nevertheless, despite the existing evidence and promising results of patient and caregiver support interventions, knowledge is lacking about their practical implementation and how these interventions can be effectively integrated into real-world clinical settings [17]. This exploration is crucial to bridge the gap between research findings and the application of evidence-based practice. Therefore, this study aims to present the findings and lessons learned from expanding, adapting, and implementing a one-on-one peer ambassador support program for patients and caregivers in a clinical setting alongside a peer training program for volunteer peer supporters.

This paper details the ongoing implementation and evaluation of a peer ambassador support program for patients and caregivers, previously developed and tested in two feasibility studies [15, 16].

## 2. Methods

The program is offered at the inpatient and outpatient clinics at the Department of Hematology, Copenhagen University Hospital, Rigshospitalet. Implementation and evaluation took place from October 2022 to January 2024 and were guided by the Grol and Wensing 5-step model of implementation [18].

### 2.1. Design: The Peer Ambassador Support Program

The ambassador support program consists of 12 weeks of one-on-one support for newly diagnosed patients or caregivers, delivered by peer ambassadors who are long-term survivors of malignant hematological diseases or their caregivers. Patients and caregivers receiving peer support are matched with an ambassador based on preferences, including support needs, diagnosis, age, gender, and life situation.

Peer support is provided approximately once a week through various contact forms, such as personal meetings, telephone calls, text messages, and video calls, based on the needs and preferences of the support recipients. There is no set time limit for the duration of each meeting.

Peer ambassadors must attend a preparatory educational course consisting of short online videos and a three-hour group session. Regular network meetings with supervision are offered to ensure a high level of safety and support for peer ambassadors.

More detailed descriptions of the intervention design for the ambassador support program and its feasibility are described elsewhere [10, 16].

#### 2.1.1. Recruitment

Peer ambassador eligibility criteria are ≥ 18 years and in complete remission for AL. Considering the varying lengths of treatment regimens and the risk of relapse, recruitment times were established to ≥ 1 year after treatment completion for patients treated for AML and/or HSCT and ≥ 2 years after treatment completion for patients with ALL. Peer ambassadors are expected to possess personal qualities such as acceptance, respect, compassion, and open-mindedness and be able to reflect on their own experiences.

The recruitment of ambassadors was conducted using two simultaneous approaches. First, physicians and program nurses, familiar with the circumstances of potential candidates, identified and approached individuals who seemed suitable for the ambassador role, providing them with program information. Second, recruitment posters and flyers were strategically placed in outpatient clinics. All applicants underwent a screening interview conducted by one of the program managers (IHN and KHN) or a program nurse to assess their readiness, personal qualities, motivations, and concerns about the role. The screening included questions such as “What are your motivations and expectations for becoming an ambassador?”, “Do you have any conditions or life circumstances that might make this role challenging?”, and “What do you think is the most important skill for an ambassador to have?” These questions were inspired by a previous study on the motivation for becoming a peer ambassador [19]. The process evaluates psychological preparedness, willingness to attend preparation and supervision, reliability, and flexibility to ensure ambassadors can provide adequate support [20]. The peer ambassadors are volunteers and receive no monetary incentive.

Patients are offered peer ambassador support if they are ≥ 18 years old and newly diagnosed with AL (AML/ALL) or plan to receive an allogeneic HSCT as part of their treatment. Patients' caregivers who are 18 years or older are eligible to participate in the program. While peer support is offered primarily early in the treatment trajectory, flexibility allows for participation later during treatment.

#### 2.1.2. Education and Support

**Figure 1:**
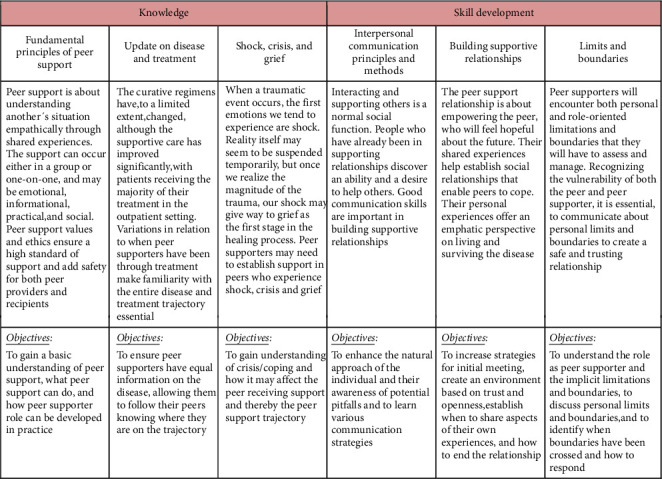
Overview of a training program for peer ambassadors.

Peer ambassadors must attend a preparatory educational course comprising six short online videos and a three-hour in-person group session. The preparatory course was developed based on findings from our previous feasibility studies and in collaboration with a trained psychologist experienced in peer support [10, 16]. The videos were developed to provide knowledge and skills, while the group sessions focused on discussing objectives, motivation, and concerns (Figure 1). Specifically, the transition from patient to ambassador is discussed, as well as considerations regarding the appropriate extent of sharing and involving their own experiences.

To ensure a high level of safety and support for peer ambassadors, we established a psychological safety net through regular network meetings with supervision every 6–8 weeks. These network meetings served as a platform for sharing experiences, addressing challenges, and providing mutual support.

### 2.2. Program Implementation: Processes and Practice Implications

The implementation and evaluation of peer ambassador support followed a multistage process guided by the Grol and Wensing implementation model which provides five step-by-step processes, guiding clinicians in successfully implementing changes into clinical practice [18]. Moreover, the practice implications that are critical to effectively implementing the peer ambassador program are described.

#### 2.2.1. Engaging a Motivated Team

The first step involved developing a plan to facilitate the change, including identifying a motivated team of clinical nurses, head nurses, and management leaders. An essential role of this team was to help identify barriers and resource constraints. Due to staff shortages, these inputs informed the decision to launch the program in only three of the five clinics. Clarity regarding the responsibilities of each step of the implementation process and defined roles and tasks was imperative.

In addition, a team of three motivated nurses was recruited as program nurses at each clinic. The program nurses were engaged for 2 h per week. In collaboration with the program managers (IHN and KHN), their roles were to introduce eligible patients and caregivers to the support program; match them with suitable ambassadors based on their support needs, diagnosis, age, and life situation; recruit and assess the new ambassadors; evaluate with participants; and facilitate ambassador education and network meetings.

#### 2.2.2. Program Flexibility

The second step involved follow-up meetings with the program and head nurses, exploring their knowledge and perceptions of peer ambassador support and identifying barriers. These discussions led to adjustments in the timing of inclusion. It was decided that peer support should be offered as soon as possible after the diagnosis of AML/ALL or during the preexamination week prior to HSCT. However, flexibility was important; while peer ambassador support should be available early in the treatment trajectory, participants should also have the option for later participation during their treatment. Program nurses would follow up with patients or caregivers, or they could opt for self-referral.

The success of the support relied on a careful match between the peer ambassador and the patient or caregiver seeking support. To facilitate meaningful connections and improve the support experience, the matching process considered the peer's diagnosis, treatment, and life situation while also allowing patients and caregivers to express preferences. If a criterion-based suitable match was not possible due to ambassador availability, patients and caregivers were asked if they would consider another ambassador. Regardless, follow-up on the match was conducted throughout the support, at the conclusion of the support, or whenever the patient or ambassador reached out for help from the team.

It was important to prioritize follow-up on each peer support trajectory during the implementation process to evaluate and provide necessary psychological support, if requested, for improving the peer ambassador support program. Furthermore, in cases where program nurses were informed about an unsuccessful match, they could refer the patient or caregiver to a new ambassador upon request. Despite the initial 12-week timeframe, ambassadors and patients or caregivers could continue their contact for as long as both parties agreed.

Each course of support was evaluated after 12 weeks and included data on satisfaction with the support and the match, its significance, knowledge and understanding (patient/caregiver), and the perspectives and consequences for the ambassador. The evaluations are described in the results section.

#### 2.2.3. Program Sustainability

The peer ambassador program materials were designed to provide standardized preparation and ensure easy dissemination and sustainability of the education program. Careful consideration to didactic aspects was given to create a well-organized, tailored, and effective educational program. For sustainability, adjustments were made to the education program in the initial step of implementation based on our experiences from the feasibility studies. These changes involved developing online videos and reducing the time of the in-person meeting, a decision deemed crucial for its time- and resource-saving benefits and providing flexibility with access to video materials from home.

Patients and caregivers who are no longer receiving support from peer ambassadors can transition to become peer ambassadors themselves if they meet the eligibility criteria. This transition enhances the program's self-sufficiency in terms of future recruitment. In addition, peer ambassadors may choose to remain involved and be matched with patients and caregivers for as long as they can contribute.

The ambassador network meetings were a key contributor to the program's success during implementation and evaluation. These meetings facilitate the exchange of experiences, provide continuous feedback and supervision, address questions and concerns, and foster relationships among ambassadors for mutual support. The program psychologist attends 2–4 times a year to deliver training and guidance on themes raised by the peer ambassadors in previous network meetings. The network meetings are facilitated by the program nurses and program managers (IHN and KHN). To date, seven ambassadors have withdrawn, primarily due to other responsibilities such as job commitments or disease relapse.

#### 2.2.4. Selection of Implementation Strategies

The third step involved selecting implementation strategies, including printed educational materials, meetings, outreach visits, e-learning, reminders, and audits and feedback [18, 21]. We provided educational materials and conducted meetings for the clinical nurses and nurse leaders, using email correspondence supplemented by on-site visits to introduce the peer ambassador support program to the involved clinics on multiple occasions. These strategies were important to ensure that all clinical sites were prepared for the implementation launch and that staff members were well-informed about the program and the impending change.

The fourth step in the Grol and Wensing model involved executing the described implementation strategies. In the days leading up to its official launch in February 2023, A3 posters and pamphlets promoting the ambassador support program were created and displayed in all patient and caregiver areas in the participating clinics.

Finally, the fifth step involved evaluating and adjusting the plan to sustain improvements, including follow-up booster sessions for clinical nurses and nurse leaders at the involved clinics. Sessions with program nurses are ongoing, held every 3–6 months, to evaluate the implementation process and make necessary adjustments.

#### 2.2.5. Ethical Considerations

This study was approved by the Copenhagen University Hospital's Ethics Committee (17–05-2017; H-17012104) and (29–07-2019; P-2019–303) adheres to the tenets of the Declaration of Helsinki.

### 2.3. Evaluation

From October 2022 to January 2023, we educated 48 peer ambassadors, comprising 36 patients and 12 caregivers. Patient ambassadors had a mean age of 58.6 years (SD: 36.6, range: 29–78 years), with a majority being men (55.6%), and nearly half (42%) held a master´s degree or higher. Caregiver ambassadors had a mean age of 63.8 (SD: 13.6, range 34–81 years), primarily females (91.7%), and 33% held a master´s degree or higher.

From February 2023 to November 2023, a total of 34 participants accepted and received peer ambassador support. The participants comprised patients (*n* = 27) and caregivers (*n* = 7) who received support from peer ambassadors (*n* = 30).  Table 1 presents the characteristics of the patients, caregivers, and peer ambassadors.

The patients had a mean age of 52.7 years. Most were females (55.6%) and married or cohabitating (70.4%). The caregivers had a mean age of 59.1 years, primarily females (85.7%), and married or cohabitating (76.7%). AML and ALL were the most frequent diagnoses, with the majority (74.1%) undergoing allogeneic stem cell transplantation.

#### 2.3.1. Satisfaction With Match

**Figure 2:**
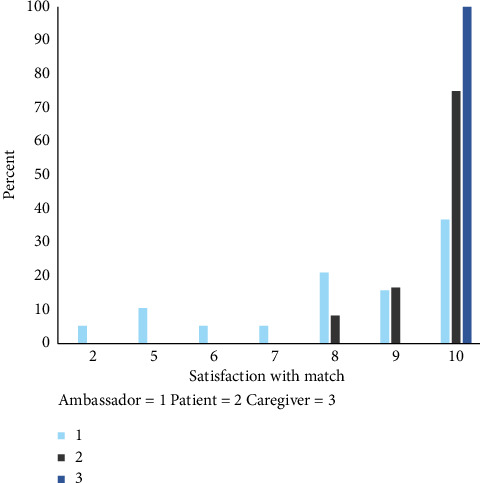
Satisfaction with a match between patient or caregiver and peer ambassador.

Overall, patients, caregivers, and ambassadors were satisfied with the match (Figure 2). Apart from one peer ambassador, who found the match with the patient too similar, triggering reflections on their disease trajectory, and requested a consultation with the psychologist, no unexpected adverse events occurred, and no peer ambassadors experienced negative consequences.

#### 2.3.2. Satisfaction With Training and Ambassador Support

Most ambassadors (84.2%) found valuable support from the educational program and the scheduled network meetings because they needed to discuss their experiences of their role as peer ambassadors, including how to manage challenges in establishing a relationship with a patient or a caregiver and how to cope when the patient´s treatment failed.

**Figure 3:**
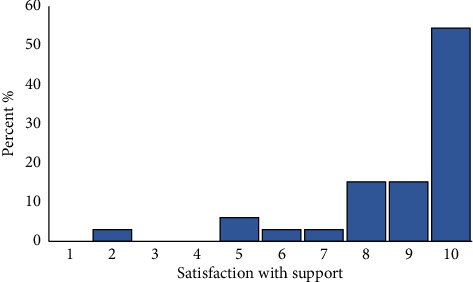
Satisfaction with support is scored from 0 to 10, with 10 representing the highest level of satisfaction and 0 representing the lowest level of satisfaction.

Satisfaction with peer support and perceived benefits:

Patients and caregivers reported the extent to which they were satisfied with the support ranging from 0–10, with 10 representing the highest level of satisfaction (Figure 3). The mean satisfaction among patients and caregivers was 8.79 (SD: 1.9), with 90.9% reporting a satisfaction level > 5 out of 10. Some, however, reported lower satisfaction due to the timing and duration of support, matching, or the ambassador's ability to take the initiative to establish communication. The majority (59%) maintained contact after 12 weeks.

**Figure 4:**
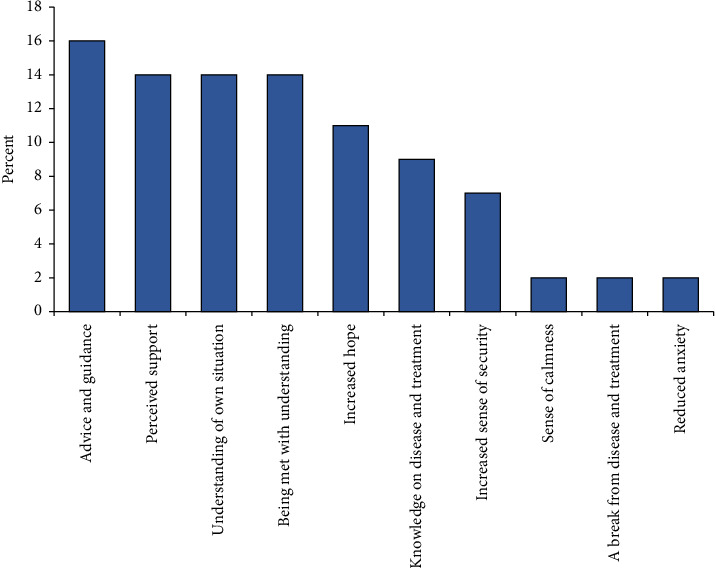
Distribution of type of support in patients and caregivers.

Most (70.5%) patients and caregivers reported that the support provided them with knowledge and new perspectives on their disease and treatment trajectory. Finally, the significance of the support was evaluated by patients and caregivers (Figure 4). It is evident that peer ambassador support provided the patients and caregivers with advice, guidance, understanding, and increased insight into their situation.

## 3. Discussion

The implementation and evaluation of peer ambassador support involved a multistage process. The Grol and Wensing implementation model provided clinicians with comprehensive, step-by-step guidance on successfully implementing changes in clinical practice [18]. Our findings demonstrate the successful adaptation and implementation of the peer ambassador support program for patients and their caregivers in clinical practice, providing valuable insights for those considering peer support programs within a hematological clinical context. However, issues related to facilitators and barriers that impact the implementation and sustainability of peer support programs in clinical practice merit discussion.

Utilizing the implementation model by Grol and Wensing [18] provided us with a comprehensive understanding of essential steps in the process and helped identify potential barriers and challenges. One key challenge was the shortage of personnel resources and time constraints, which led program nurses to encounter difficulties in effectively executing tasks and adapting routines for recruiting, matching, and evaluating during a busy day in the clinic. A recent systematic review [22] found that integrating volunteers into healthcare services can impose an additional burden on healthcare professionals. It suggests that a designated volunteer coordinator may be a solution to manage the coordination of volunteers, including matching and recruitment [22]. Therefore, ensuring adequate resources is crucial to successfully implementing and sustaining volunteer peer support.

Conducting network meetings, training courses, and recruitment within the hospital setting presents both challenges and opportunities. The knowledge that healthcare professionals have about their patients and caregivers can enhance the recruitment of new peer ambassadors and ensure more successful matching, a critical factor for the success of the peer support program. This level of personal and medical insight would be challenging to achieve if the ambassador support program were located outside the hospital or within a patient organization. We found that program nurses and physicians received positive, spontaneous feedback from patients and caregivers during their hospital visits, validating the program's success. In addition, program nurses needed to participate in the network meetings, ensuring effective communication and collaboration between peer ambassador supporters and healthcare professionals during implementation, and increasing ownership among nurses. Furthermore, nurses can identify issues in the clinical setting, such as a lack of contact between peer ambassador supporters and recipients, enabling timely intervention. However, the provision of peer support in the ambassador program mainly occurs outside the hospital setting through telephone, text messages, and in-person meetings held in public, at home, or at the hospital, depending on preferences and practicality. Notably, a qualitative study on peer support centers in Norway [23] found that patients and caregivers valued the opportunity to meet peer supporters at a community cancer center, where the atmosphere was more relaxed compared to the hospital setting.

Despite employing diverse recruitment strategies, most of the peer ambassadors were enlisted through physicians and nurses in the clinics. While recruiting through clinicians has not been recommended in previous studies [24], this approach demonstrated success in recruiting and evaluating their readiness. In addition, being recognized for the skills necessary for peer support likely played a positive role in motivating and fostering the willingness to participate as peer ambassadors [19]. However, this may have contributed to a less diverse group, with nearly half of the ambassadors being socioeconomically advantaged. This is relevant for the recruitment process when implementing ambassador support, as targeted efforts can be made to ensure a diverse range of characteristics among the ambassadors.

Implementing and evaluating processes are often challenging, particularly when multiple sites are engaged [25]. A distinctive advantage of our implementation process is its focus on a single hospital site with three clinics, reducing complexities, enhancing the likelihood of success, and fostering ownership among healthcare professionals and peer ambassadors. However, certain limitations must be acknowledged. Similar to previous findings, most participants in our program are women [26]. Effective strategies to engage individuals from diverse backgrounds, including ethnicities and family caregivers, are important for ensuring that the program reflects the diversity of the patient and caregiver population. Moreover, to enhance recruitment and ensure the program's sustainability, collaborating with patient cancer community organizations could be beneficial. These organizations may help reach potential participants, raise awareness about the program to a broader audience, and maintain engagement over time [27]. A limitation is that our evaluation exclusively considered the perspectives of peer ambassador supporters and recipients, omitting input from program nurses and physicians. Exploring their viewpoints could have provided more comprehensive insights into the facilitators and challenges of the implementation process.

## 4. Clinical Implications

Peer ambassador support should complement existing supportive care services available to patients undergoing treatment for hematological malignancies and their caregivers. Implementing and evaluating peer ambassador support in clinical practice require an infrastructure where nurses play a pivotal role in recruiting, training, supervising, maintaining peer ambassadors, and conducting program evaluations. Given that nurses are integral to supportive care in cancer, they will play an important role in the implementation and sustainability of peer ambassador support. Consistent with our previous studies, the findings of this paper indicate the relevance of peer ambassador support early in the treatment trajectory for malignant hematological diseases. It highlights the need for a more holistic clinical approach, integrating peer ambassador support as a component of supportive care during treatment and survivorship across the disease and treatment continuum.

This paper contributes novel insights into peer ambassador support for patients and caregivers in a clinical hematological setting. The peer ambassador support program is evidence-based, described in detail, and thus easy to implement in clinical practice. Finally, this paper provides valuable knowledge for implementing future initiatives involving peer ambassador support, with potential applicability to patients with life-threatening illnesses and their caregivers.

## 5. Conclusion

This paper demonstrates that peer ambassador support to patients undergoing treatment for a hematological disease and their caregivers is implementable in a clinical setting. Key conclusions about the lessons learned include the following:

• Securing management support before implementing it into clinical practice is essential. This support includes allocating operational costs and necessary staff resources, such as a peer support coordinator and program nurses.
• Strategies for recruiting volunteer peer supporters and retention tactics, such as education and regular network meetings for volunteers, should be considered.
• When integrating evidence into clinical practice, it is crucial to tailor and adjust the intervention and training program to fit the clinical context, incorporating continuous input and adjustments.
• Emphasizing the need for convenient availability of peer supporters and ensuring flexibility and sustainability of program materials are essential for successful implementation.

Ultimately, researchers and clinicians must consider the challenges and benefits of implementing evidence-based knowledge into clinical practice. We highly recommend implementing strategies to guide the process and address the time-consuming provision of necessary resources.

## Figures and Tables

**Table 1:** Demographic and clinical characteristics.

Characteristic	Patients (*n* = 27)	Caregivers (*n* = 7)	Peer ambassadors (*n* = 30)
Gender, female, *n* (%)	15 (55.6)	6 (85.7)	18 (60.0)
Age, mean (SD)	52.7 (14.1)	59.1 (10.1)	54.5 (14.2)
Range	21–72	45–68	22–72
Categories, *n* (%)			
18–39	5 (18.5)		4 (13.3)
40–65	13 (48.2)	4 (57.1)	16 (53.3)
65 or older	9 (33.3)	3 (42.9)	10 (33.3)
Unknown			
Marital status, *n* (%)			
Married or cohabitating	19 (70.4)	7 (100)	23 (76.7)
Single, separated, divorced, or widowed	8 (29.6)		6 (20.0)
Unknown			1 (3.3)
Caregiver relation, *n* (%)			
Spouse		6 (85.7)	
Parent		1 (14.3)	
Diagnosis, *n* (%)			
Acute leukemia	16 (59.3)	5 (71.4)	17 (56.7)
Chronic leukemia	3 (11.1)	2 (28.6)	
Lymphoma	1 (3.7)		6 (20.0)
Myelodysplastic syndrome	6 (22.2)		7 (23.3)
Other	1 (3.7)		
Time from diagnosis, mean (SD)			4.23 (2.3)
Range			2–10
Allogeneic stem cell transplant, *n* (%)	20 (74.1)	5 (71.4)	72 (90%)

*Note:* In caregiver peer ambassadors, time from diagnosis is the time from the patients' diagnosis.

## Data Availability

The data that support the findings of this study are available on request from the corresponding author. The data are not publicly available due to privacy or ethical restrictions.
